# CXCR6 marks a novel subset of T-bet^lo^Eomes^hi^ natural killer cells residing in human liver

**DOI:** 10.1038/srep26157

**Published:** 2016-05-23

**Authors:** Kerstin A. Stegmann, Francis Robertson, Navjyot Hansi, Upkar Gill, Celeste Pallant, Theodoros Christophides, Laura J. Pallett, Dimitra Peppa, Claire Dunn, Giuseppe Fusai, Victoria Male, Brian R. Davidson, Patrick Kennedy, Mala K. Maini

**Affiliations:** 1 Division of Infection and Immunity and Institute of Immunity and Transplantation, UCL, London, UK; 2 Department of Surgery and Interventional Science, UCL, Royal Free Hospital London, London, United Kingdom; 3 Centre for Digestive Diseases, Blizard Institute, Bart’s and the London School of Medicine and Dentistry, London, UK

## Abstract

Natural killer cells (NK) are highly enriched in the human liver, where they can regulate immunity and immunopathology. We probed them for a liver-resident subset, distinct from conventional bone-marrow-derived NK. CXCR6+ NK were strikingly enriched in healthy and diseased liver compared to blood (p < 0.0001). Human hepatic CXCR6+ NK had an immature phenotype (predominantly CD56^bright^CD16−CD57−), and expressed the tissue-residency marker CD69. CXCR6+ NK produced fewer cytotoxic mediators and pro-inflammatory cytokines than the non-liver-specific CXCR6− fraction. Instead CXCR6+ NK could upregulate TRAIL, a key death ligand in hepatitis pathogenesis. CXCR6 demarcated liver NK into two transcriptionally distinct populations: T-bet^hi^Eomes^lo^(CXCR6−) and T-bet^lo^Eomes^hi^(CXCR6+); the latter was virtually absent in the periphery. The small circulating CXCR6+ subset was predominantly T-bet^hi^Eomes^lo^, suggesting its lineage was closer to CXCR6− peripheral than CXCR6+ liver NK. These data reveal a large subset of human liver-resident T-bet^lo^Eomes^hi^ NK, distinguished by their surface expression of CXCR6, adapted for hepatic tolerance and inducible anti-viral immunity.

The liver has a highly specialised immunological composition, enriched for a number of innate effector cells. NK cells are the most prevalent cell type amongst human intrahepatic leukocytes, accounting for around 30–40% of the total^1^. Accumulating data have underscored the key role that this large population can play in balancing hepatic tolerance and immunity^2^. In the hepatitis B virus (HBV)-infected liver they maintain cytotoxic capacity but have impaired non-cytolytic function, with reduced capacity to produce IFNγ^3^. We previously demonstrated that liver NK cells can upregulate the death ligand TRAIL, giving them the capacity to kill hepatocytes that express the death-inducing receptor TRAIL-R2 in chronic hepatitis B (CHB), thereby mediating an antiviral effect at the expense of liver damage^3^,^4^. Unlike in the mouse, human NK cells express minimal TRAIL in the healthy liver but can be triggered to express it by endogenous^4^ or therapeutic^5^ IFNα. An IFN-induced expansion of TRAIL-expressing NK cells with the potential to kill infected hepatocytes was likewise demonstrated in another hepatotropic viral infection, hepatitis C^6^,^7^. In addition to this important role in killing virally infected hepatocytes and driving viral hepatitis, we recently showed that liver NK cells have the capacity to regulate antiviral T cells^8^. In line with emerging data for a role for NK cells as rheostats modulating T cell immunopathology^9^,^10^, we reported that NK cells in patients with CHB can selectively kill HBV-specific T cells. This was partially mediated through the TRAIL pathway, with a high frequency of intrahepatic HBV-specific T cells expressing the TRAIL-R2 death receptor not normally seen on T cells^8^. In addition, NK cells may limit liver fibrosis by interacting with and killing hepatic stellate cells through a number of receptor ligand pairs including TRAIL^11^. Thus diverse protective and pathogenic roles are emerging for liver NK cells, emphasizing the importance of understanding more about their origin and diversity.

Exciting progress has been made recently in our understanding of subsets of NK cells present within the mouse liver, with the demonstration of a CXCR6 expressing liver NK cell population capable of mediating recall responses to previously encountered pathogens or haptens^12^,^13^. Further work has revealed that a subset of murine liver NK cells constitute a separate lineage to the classical bone marrow-derived NK cells. DX5−TRAIL+ murine liver NK cells expressing CD49a and CXCR6 were found to have a transcriptional profile distinct from the other DX5+TRAIL– subset in the liver^13^,^14^,^15^. The DX5−CD49a+ subset was shown to be liver-resident using parabiosis experiments, whereas the remaining DX5+CD49a− liver NK cells were exchanged between parabionts^13^. Further experiments revealed that the liver-resident NK cell subset was not derived from bone-marrow precursors like conventional NK cells, but could instead originate from hepatic progenitor cells, in line with their dominance in the fetal liver^13^. Differential dependence on transcription factors of these two murine liver NK cell subsets confirmed their distinct lineages^14^,^15^; in Eomes knockout mice the development of conventional NK cells in liver and spleen was markedly reduced whereas the liver-resident DX5−TRAIL+ population was not affected. T-bet knockout mice on the other hand, had a preferential reduction in DX5−TRAIL+CXCR6+ liver NK cells. Ectopic expression of T-bet in liver progenitors repressed Eomes expression and forced the development of Eomes– liver-resident NK cells.

Here we therefore interrogated human liver NK cells to see if, as in murine studies, we could distinguish a liver-resident subset with a unique transcription factor profile.

## Results and Discussion

### CXCR6+ NK cells are enriched in the healthy and diseased human liver

**Figure 1:**
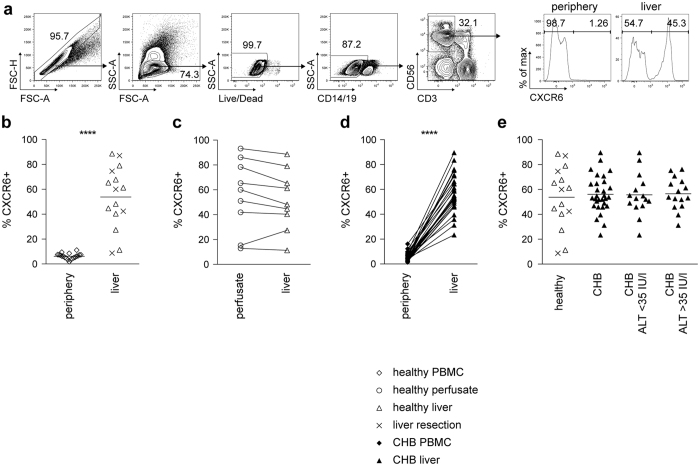
CXCR6+ NK cells are enriched in the liver in health and disease. (**a**) Gating strategy for identification of NK cells (singlets, live cells, CD14−, CD19−, CD3−, CD56+ cells) using multicolor flow cytometry and representative histograms of CXCR6 staining of NK cells from periphery and liver. (**b**) Summary frequencies of CXCR6+ NK cells from PBMC (n = 20), and liver (n = 15), comprising biopsies obtained from healthy donor livers (n = 10) or healthy margins of resections for colorectal metastases (n = 5). (**c**) Direct comparison of CXCR6 frequencies of NK cells isolated from perfusion liquid and liver tissue from the same healthy controls (n = 9). (**d**) Summary of CXCR6 frequencies of NK cells from paired PBMC and liver biopsies from patients with CHB (n = 30). (**e**) Summary of CXCR6 expression on NK cells from healthy liver (n = 15), compared to liver tissue from patients with CHB (n = 30). CHB patients were additionally divided by ALT (low ALT < 35 IU/l, n = 15; high ALT > 35 IU/l, n = 15). Diagrams show individual data for healthy controls and CHB. P-values indicated in each diagram. Mann-Whitney U-test (**b**), Kruskal-Wallis with Dunn’s multiple comparison test (**e**) and paired Wilcoxon test (**c**,**d**) were applied. ****p-value < 0.0001.

To study the composition of the NK cell compartment in human liver we took advantage of access to the following valuable collection of samples: pre-implant biopsies and perfusion liquid from cadaveric donor livers prior to transplantation, resection tissue from healthy liver surrounding colorectal metastases, and biopsies with paired blood from patients with CHB. NK cells in periphery or intrahepatic leukocytes (IHL) were identified by the gating strategy shown in Fig. 1a; in brief, after exclusion of cell aggregates, gating on lymphocytes and subsequent exclusion of dead cells, CD14+ and CD19+ cells, NK cells were identified as CD3−CD56+.

Intrahepatic NK cells were highly enriched for the expression of the surface chemokine receptor CXCR6, which was only found on a small proportion of peripheral NK cells (Fig. 1a,b). Paired perfusion liquid and pre-implant biopsies obtained from the same cadaveric donor livers had similarly high frequencies of CXCR6+NK cells (Fig. 1c). CXCR6+ NK cells are therefore present within the liver vasculature, compatible with the reported expression of the ligand CXCL16 by liver sinusoidal populations^16^,^17^. Sampling of a cohort of patients with CHB, from whom we had the opportunity to obtain paired blood and intrahepatic samples, similarly showed a striking selective increase in CXCR6+ NK cells within the hepatic compartment in all cases (mean 56%, Fig. 1d). The enrichment of CXCR6 expressing NK cells seen in the healthy liver was therefore maintained in diseased livers, even in the presence of HBV-related liver inflammation (measured by alanine transaminase, ALT, Fig. 1e), which is characterised by a large lymphocytic infiltrate^18^.

Thus we describe a surface marker defining a subset of NK cells that is markedly enriched in the liver of healthy donors, and maintained at similarly high frequencies in patients with a variable degree of HBV-related liver inflammation. The proportion of CXCR6+ NK cells varied widely amongst both healthy and HBV-infected livers. The drivers of this variation in CXCR6+ NK cell hepatic accumulation remain to be determined; their frequency could not be attributed to any of the factors we examined (age, CMV serostatus; in CHB cohort: HBV viraemia, eAg status, ALT).

### CXCR6+ liver NK cells have an immature, tissue-resident phenotype

**Figure 2:**
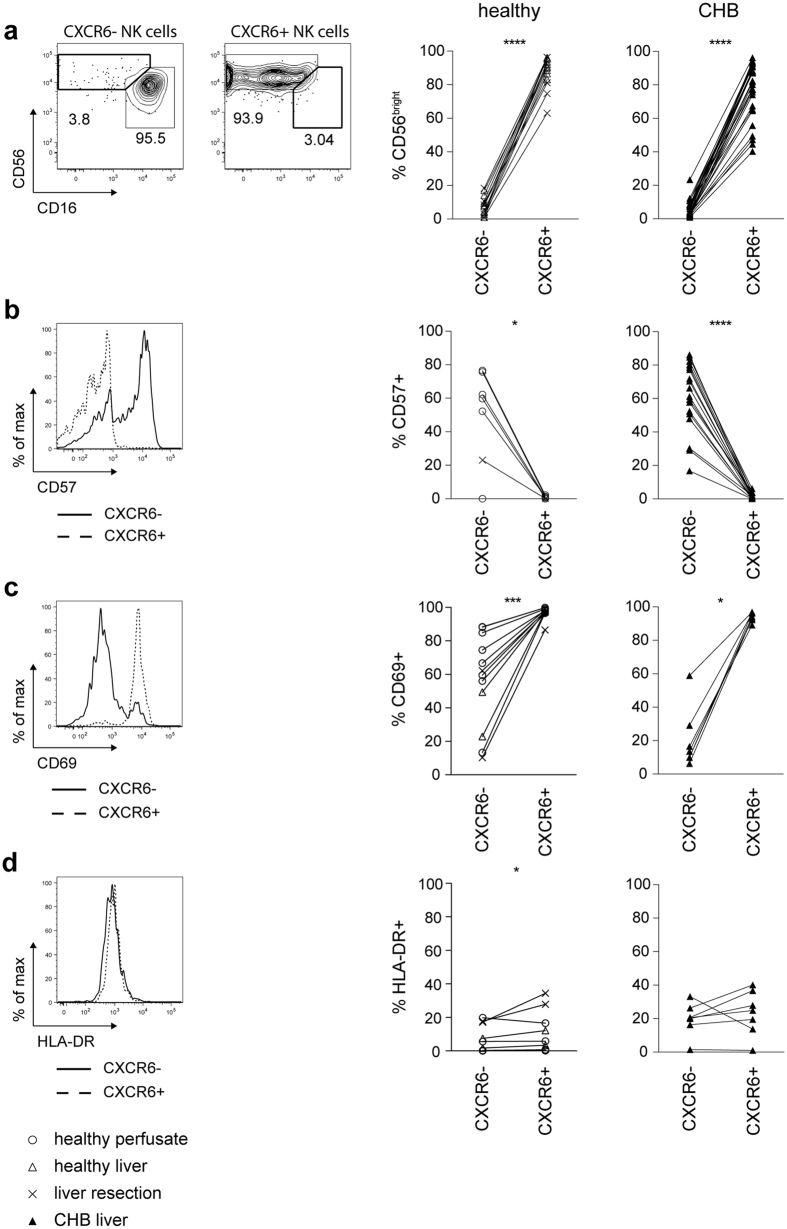
Intrahepatic CXCR6+ NK cells have an immature phenotype and express high levels of CD69. (**a**) Representative contour plots for identification of CD56^bright^ and CD56^dim^ NK cells based on CD16/CD56 expression within intrahepatic CXCR6− and CXCR6+NK cells. Summary of frequency of CD56^bright^ NK cells in intrahepatic CXCR6− and CXCR6+ NK cells of liver tissue of healthy controls (n = 15) and patients with CHB (n = 30). (**b**) Representative histogram overlay of CD57 expression on intrahepatic CXCR6− and CXCR6+ NK cells. Summary of frequency of CD57 expressing intrahepatic CXCR6− and CXCR6+NK cells of healthy controls (perfusion liquid n = 6; liver tissue n = 1) and patients with CHB (n = 18). (**c**) Representative histogram overlaying CD69 expression on intrahepatic CXCR6− and CXCR6+ NK cells. Summary of frequency of CD69 expressing intrahepatic CXCR6− and CXCR6+ NK cells of healthy controls (fresh perfusion liquid n = 2; frozen perfusion liquid n = 6; liver tissue n = 4) and of patients with CHB (n = 7). (**d**) Representative histogram overlaying HLA-DR expression on intrahepatic CXCR6− and CXCR6+ NK cells. Summary of frequency of HLA-DR expressing intrahepatic CXCR6− and CXCR6+ NK cells of healthy controls (fresh perfusion liquid n = 2; frozen perfusion liquid n = 6; liver tissue n = 4) and patients with CHB (n = 7). Paired Wilcoxon test was applied for all figures. *p-value < 0.05, ***p-value < 0.001, ****p-value < 0.0001.

We then compared the surface marker expression of CXCR6+ and CXCR6− fractions of intrahepatic NK cells to better define their characteristics. CXCR6+ NK cells in the healthy human liver were predominantly CD56^bright^CD16^low/intermediate^, whereas the CXCR6− subset was largely CD56^dim^CD16+ (Fig. 2a). In the HBV-infected liver, CXCR6+ NK cells remained enriched for the immature markers CD56^bright^CD16^low/intermediate^ (compared to the CXCR6− subset), although to a more variable degree than in the healthy liver (Fig. 2a). CXCR6+ NK cells in the liver consistently lacked expression of CD57 (Fig. 2b), a marker that has been associated with NK cell maturity/antigen exposure^19^,^20^. All CD57+ staining on NK cells in healthy or diseased liver segregated with the CXCR6− subset (Fig. 2b). This is concordant with findings in the murine liver, where the liver-resident population of DX5− NK cells has a more immature phenotype^13^,^15^.

Expression of CD69 has recently been described to be a distinguishing feature of human tissue-resident memory T cells^21^, inhibiting sphingosine-1-phosphate (S1P1) receptor function to regulate peripheral T cell retention^22^,^23^,^24^; we therefore examined its expression on liver NK cells. In line with the concept that the CXCR6+ NK cells may be liver-resident, 95% co-expressed CD69 directly *ex vivo* (Fig. 2c). Although commonly used as a marker of activation, in this setting CD69 was more likely reflective of tissue residency, since CXCR6+ NK cells were not similarly enriched for other markers of activation such as HLA-DR (Fig. 2d).

### CXCR6 marks a subset of hepatic NK cells functionally adapted to the liver niche

**Figure 3:**
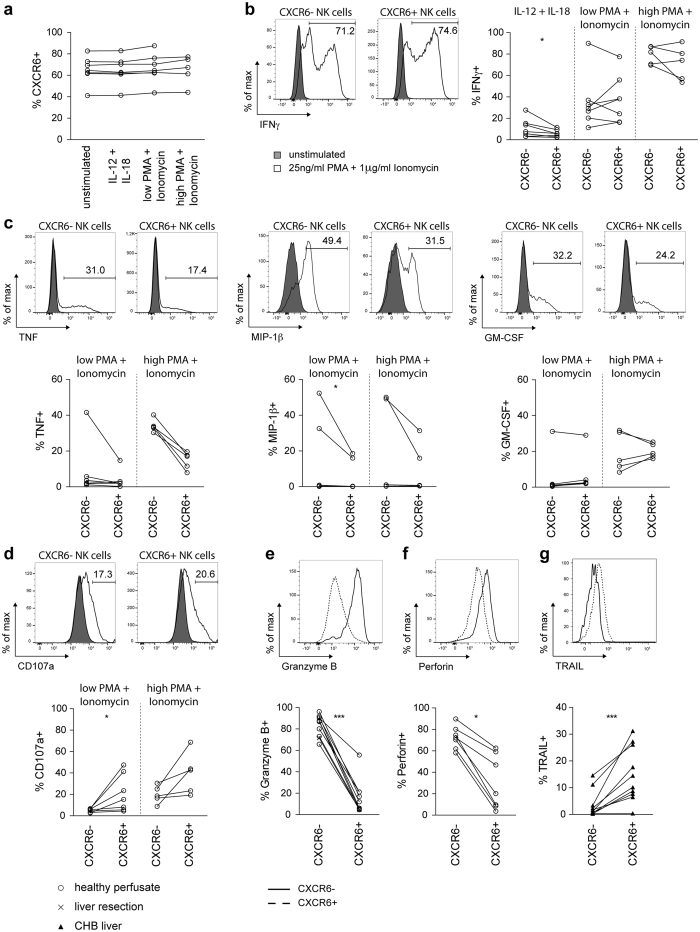
Functional capacity of CXCR6− and CXCR6+ intrahepatic NK cells. (**a**) Summary of percentage of NK cells isolated from perfusates expressing CXCR6 with or without stimulation with IL-12/IL-18 (5 ng/ml, 50 ng/ml) or PMA and Ionomycin at low (3 ng/ml, 100 ng/ml) or high (25 ng/ml, 1 μg/ml) dose (n = 7; 7; 7; 5). (**b**) Representative histograms overlaying IFNγ production of unstimulated, and high dose PMA/Ionomycin stimulated, for intrahepatic CXCR6− and CXCR6+ NK cells. Summary of IFNγ production by CXCR6− and CXCR6+ NK cells from healthy liver perfusates with stimuli as indicated (n = 7; 7; 5), after subtraction of the unstimulated control. (**c**) Representative histograms overlaying TNF, MIP-1β and GM-CSF production of unstimulated, and high dose PMA/Ionomycin stimulated, intrahepatic CXCR6− and CXCR6+ NK cells. Summary of TNF, MIP-1β and GM-CSF production by CXCR6− and CXCR6+ NK cells from healthy liver perfusates with stimuli as indicated (n = 7; 5), after subtraction of the unstimulated control. (**d**) Representative histograms overlaying CD107a expression of unstimulated, and high dose PMA/Ionomycin stimulated, intrahepatic CXCR6− and CXCR6+NK cells. Summary of CD107a expression by CXCR6− and CXCR6+NK cells from healthy liver perfusates with stimuli as indicated (n = 7; 5), after subtraction of the unstimulated control. (**e**) Representative histogram overlaying *ex vivo* granzyme B expression in intrahepatic CXCR6− and CXCR6+ NK cells. Summary of frequency of granzyme B expressing intrahepatic CXCR6− and CXCR6+ NK cells from healthy controls (perfusion liquid n = 9; liver tissue n = 1). (**f**) Representative histogram overlaying *ex vivo* perforin expression in intrahepatic CXCR6− and CXCR6+ NK cells. Summary of frequency of perforin expressing intrahepatic CXCR6− and CXCR6+ NK cells from healthy controls (perfusion liquid n = 7). (**g**) Representative histogram overlaying TRAIL expression on intrahepatic CXCR6− and CXCR6+ NK cells from a patient with CHB. Summary of frequency of TRAIL expressing intrahepatic CXCR6− and CXCR6+ NK cells from patients with CHB (n = 10). Kruskal-Wallis with Dunn’s multiple comparison test (A) and Paired Wilcoxon test (B–G) was applied. *p-value < 0.05, ***p-value < 0.001.

To examine their effector function, liver-infiltrating lymphocytes from perfusates of healthy livers were stimulated with IL-12/IL-18 or PMA/ionomycin, since negligible cytokine production was observed *ex vivo*. Short-term stimuli (4 hours) did not alter surface CXCR6 expression (Fig. 3a). Although strong stimulation with low or high-dose PMA/ionomycin induced a large and variable percentage of both CXCR6+ and CXCR6− fractions to produce IFNγ, the more physiological stimulus of IL-12/IL-18 induced less IFNγ from the CXCR6+ than the CXCR6− fraction (Fig. 3b). Additional pro-inflammatory cytokines/chemokines TNF and MIP-1β also tended to be produced by lower percentages of CXCR6+ than CXCR6− intrahepatic NK cells following low or high-dose PMA/ionomycin, whilst GM-CSF production showed no clear difference (Fig. 3c; IL-12/IL-18 failed to elicit these cytokines). CXRC6+ intrahepatic NK cells were more likely to degranulate upon PMA/ionomycin stimulation than their CXCR6− counterparts (Fig. 3d); however *ex vivo* granzyme B and perforin levels revealed that the CXCR6+ fraction have strikingly less constitutive cytotoxic capacity (Fig. 3e,f).

NK cells can also mediate perforin-independent cytotoxicity, which is likely to be particularly relevant in the liver since hepatocytes are relatively resistant to perforin/granzyme cytotoxicity^2^. Cytotoxicity through death ligands such as TRAIL has been shown to be critical for liver damage in several murine models as well as HBV and HCV^2^. Murine liver-resident NK cells (DX5−) express high levels of TRAIL^25^ whereas NK cells express negligible TRAIL in the healthy human liver^26^. In contrast, in HBV-related liver inflammation we have described an expansion of TRAIL-expressing NK cells that make an important contribution to disease pathogenesis^4^,^8^. Co-staining NK cells from HBV-infected livers with CXCR6 and TRAIL established that TRAIL induction was largely restricted to the CXCR6+ fraction (Fig. 3g).

### The liver contains a distinct T-bet^lo^ Eomes^hi^ NK cell subset identified by CXCR6

**Figure 4:**
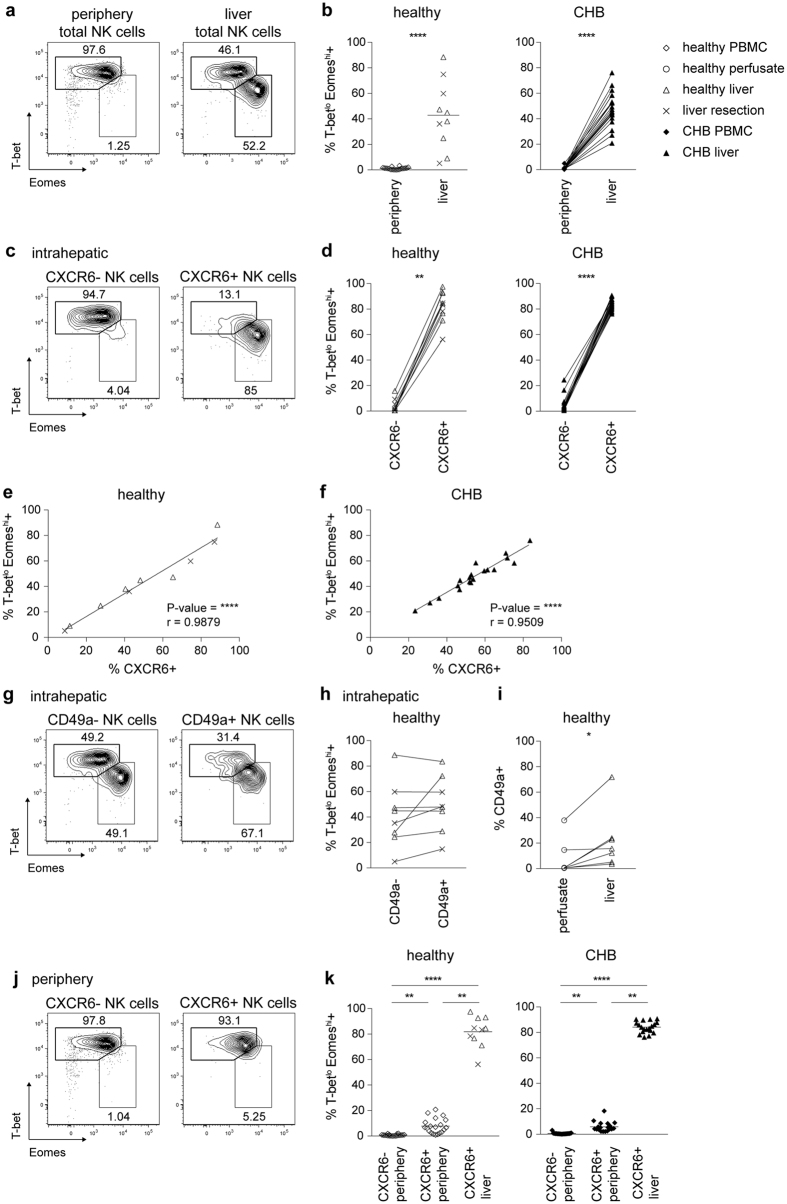
The liver contains a distinct T-bet^lo^Eomes^hi^ NK cell subset identified by CXCR6, but not CD49a expression. (**a**) Representative contour plots for identification of T-bet^hi^Eomes^lo^ and T-bet^lo^Eomes^hi^ subsets of total NK cells from periphery and liver tissue. (**b**) Summary of frequency of T-bet^lo^Eomes^hi^ cells of total NK cells in PBMC (n = 20) and liver tissue of healthy controls (n = 10), and paired PBMC and liver biopsies from patients with CHB (n = 19). (**c**) Representative contour plots for identification of T-bet^lo^Eomes^hi^ and T-bet^hi^Eomes^lo^ subsets in intrahepatic CXCR6− and CXCR6+ NK cells. (**d**) Summary of frequency of T-bet^lo^Eomes^hi^ cells among intrahepatic CXCR6− and CXCR6+ NK cells of liver tissue of healthy controls (n = 10) and patients with CHB (n = 19). (**e**,**f**) Correlation of frequency of T-bet^lo^Eomes^hi^ and CXCR6 expression of NK cells from liver tissue of healthy controls ((**e**) n = 10) and liver biopsies from patients with CHB ((**f**) n = 19). (**g**) Representative contour plots for identification of T-bet^lo^Eomes^hi^ and T-bet^hi^Eomes^lo^ subsets among intrahepatic CD49a− and CD49a+ NK cells. (**h**) Summary of frequency of T-bet^lo^ Eomes^hi^ cells among intrahepatic CD49a− and CD49a+ NK cells of healthy liver tissue (n = 8). (**i**) Summary of frequency of CD49a expression on intrahepatic NK cells from paired perfusion liquid and tissue from pre-implant donor livers (n = 7). (**j**) Representative contour plots for identification of T-bet^lo^Eomes^hi^ and T-bet^hi^Eomes^lo^ subsets among peripheral CXCR6− and CXCR6+ NK cells. (**k**) Summary of frequency of T-bet^lo^Eomes^hi^ cells among peripheral CXCR6− and CXCR6+ NK cells (n = 20) and intrahepatic CXCR6+NK cells of liver tissue of healthy controls (n = 10); and of peripheral CXCR6− and CXCR6+ NK cells and paired intrahepatic CXCR6+ NK cells of patients with CHB (n = 19). P-values and r indicated in each diagram. Mann-Whitney U-test ((**b**) left), Kruskal-Wallis with Dunn’s multiple comparison test ((**k**) left), Friedmans with Dunn’s multiple comparision test ((**k**) right), paired Wilcoxon test ((**b**) right, (**d**,**h**,**i**)) and Spearman’s correlation (**e**,**f**) were applied. *p-value < 0.05, **p-value < 0.01, ****p-value < 0.0001.

Differential expression of the transcription factors T-bet and Eomes has been shown to instruct the development of distinct lineages of NK cells in mice, with murine liver-resident NK cells having a unique T-bet^hi^Eomes^lo^ profile^13^,^14^,^15^. Instead in humans, peripheral NK cells have recently been reported to be predominantly T-bet^hi^Eomes^lo ^27, confirmed in the donors we sampled (Fig. 4a); however, a distinct profile of T-bet/Eomes usage for human liver NK cells has not been defined. We found that intrahepatic NK cells could be divided into two populations according to their expression of T-bet and Eomes; in addition to the T-bet^hi^Eomes^lo^ staining characteristic of peripheral NK cells, a mean of 48% of hepatic NK cells had the converse T-bet^lo^Eomes^hi^ profile (Fig. 4a,b). The percent of T-bet^lo^Eomes^hi^ NK cells was similarly variable in healthy and HBV-infected livers, ranging from 5% to 88% percent of total intrahepatic NK cells; in contrast, NK cells with this transcription factor profile were consistently barely detectable in the circulation of healthy donors or patients with CHB (Fig. 4b).

Further analysis revealed that the expression of CXCR6 on intrahepatic NK cells was a robust surface marker to predict their transcription factor profile. The majority of CXCR6+ liver NK cells were T-bet^lo^Eomes^hi^, whereas liver NK cells lacking CXCR6 mostly had the converse pattern of T-bet^hi^Eomes^lo^, with a mean of only 4% being T-bet^lo^Eomes^hi^ (Fig. 4c,d). The link between surface CXCR6 and intranuclear Eomes was underscored by the very strong correlation between the percentage expression of these two markers for intrahepatic NK cells within healthy and HBV-infected livers (Fig. 4e,f). This capacity to dissect liver NK cells into discrete T-bet^hi^Eomes^lo^ and T-bet^lo^Eomes^hi^ subsets was unique to CXCR6 and not a property of CD49a, another marker recently described to define a population of human liver NK cells^28^ (Fig. 4g,h). However we did confirm that CD49a+NK cells were more enriched in liver parenchyma than liver vasculature (Fig. 4i), albeit at lower overall frequencies than CXCR6+ NK cells.

To further probe whether CXCR6 defined a population of liver-resident NK cells, we next examined the small fraction of circulating NK cells that expressed CXCR6. CXCR6+ peripheral NK cells had a transcription factor profile much more analogous to that of CXCR6− peripheral NK cells than to their CXCR6+ intrahepatic counterparts. CXCR6+ peripheral NK cells were mainly T-bet^hi^Eomes^lo^ like most peripheral NK cells (Fig. 4j,k), in contrast to the predominant T-bet^lo^Eomes^hi^ profile amongst the much larger fraction of CXCR6+ NK cells in the liver. The fact that the small population of CXCR6 expressing NK cells detectable in the periphery did not share their transcription factor usage with their liver counterparts, even in the dynamic situation of HBV-related liver inflammation, supports the concept of hepatic CXCR6+ NK cells being liver-resident. Liver CXCR6+ T-bet^lo^Eomes^hi^ NK cells could either be a separate lineage derived from hepatic progenitors^13^ and/or might be recruited from the periphery^29^ and acquire their liver-specific profile by *in situ* differentiation in response to local microenvironmental cues.

In conclusion, we demonstrate that CXCR6 dissects human liver NK cells into two discrete subsets. The CXCR6+ fraction displays a predominantly CD56^bright^CD16−CD57− phenotype, capable of expressing TRAIL in diseased liver, analogous to the DX5−TRAIL+CXCR6+liver-resident NK cell population in the mouse. We show that CXCR6 expression marks a novel, sizeable population of T-bet^lo^Eomes^hi^ NK cells within the human liver that are barely detectable in the periphery. The chemokine CXCL16, the ligand for CXCR6, is strongly expressed in the sinusoids of human liver^16^,^17^, providing a mechanism for hepatic retention of CXCR6 expressing lymphocytes. The uniform co-expression of CD69 by CXCR6+ NK cells may also confer tissue residency through its capacity to prevent lymphocyte egress by counteracting S1P1^23^,^24^,^30^. Expression of CD69 is now well accepted as a marker of tissue-resident T cells^21^; our data reveal its applicability to denote resident NK cells.

The liver-resident CXCR6+ subset of NK cells is well-adapted to the tolerogenic liver niche, since in the healthy liver they have low expression of cytotoxic mediators and pro-inflammatory cytokines. However their capacity to express TRAIL in response to a pathogen challenge renders CXCR6+ T-bet^lo^Eomes^hi^ NK cells capable of regulating liver immunopathology in viral hepatitis by TRAIL-dependent interactions with hepatocytes and antiviral T cells^2^. We therefore propose that the population of CXCR6+ T-bet^lo^Eomes^hi^ NK cells residing in the human liver serves a key role as an inducible defence mechanism, eliminating infected or transformed cells and curtailing excessive T cell responses. In the setting of a persistent infection such as HBV, their homeostatic effects can become detrimental, contributing to liver pathology and preventing an adequate antiviral T cell response. The capacity to distinguish this population of T-bet^lo^Eomes^hi^ NK cells by their surface expression of CXCR6 will expedite future studies to further explore their role in liver immunity and disease.

## Methods

### Patients and controls

This study was approved by the local ethical boards of the Central and North West London NHS Trust, The Royal London Hospital and The Royal Free Hospital, with all participants giving written informed consent. Blood and paired surplus tissue from liver biopsies of patients with untreated CHB were obtained from the Royal London Hospital. Healthy margins of metastatic tumor resections of 5 patients were obtained from the Royal Free Hospital. Perfusion liquid of 18 liver transplantations, 9 with paired pre-implant liver biopsies (and one unpaired biopsy), taken from healthy cadaveric livers to be used for transplantation, were obtained from the Royal Free Hospital. Blood was taken from 20 healthy controls who were recruited at University College London. Experiments were carried out in accordance with approved guidelines. Demographic and clinical characteristics of donors are summarised in Table 1.

### Sample preparation

PBMC were isolated from heparinized blood by density centrifugation using a standard Ficoll density centrifugation method. Liquid used to transport and perfuse healthy cadaveric livers prior to transplantation (perfusates) was centrifuged to concentrate the volume, before Ficoll density isolation of liver intravascular leukocytes. To obtain intrahepatic leukocytes from liver tissue, biopsy or resection fragments were mechanically dissociated before passing through a 70 μm cell strainer. Intrahepatic leukocytes isolated from liver biopsies were used immediately whereas those isolated from larger liver tissue were isolated with an extra Percoll density centrifugation step before experimentation.

### Antibodies used for characterization of intrahepatic NK cells

The following anti-human antibodies were used in this study: anti-CD3-BV605 (clone OKT3, Biolegend), anti-CD3-BUV395 (clone UCHT1, BD Bioscience), anti-CD14-V500 (clone M5E2, BD Bioscience), anti-CD19-BV510 (clone SJ25C1, BD Bioscience), anti-CD56-PE-Cy7 (clone NCAM16.2, BD Bioscience), anti-CXCR6-PE or anti-CXCR6-APC (clone K041E5, Biolegend), anti-CD57-AF488 (clone TB01, eBioscience), anti-TRAIL-BV421 (clone RIK-2, BD Bioscience), anti-CD49a-FITC (clone TS2/7, Biolegend), anti-HLA-DR V500 (clone G46-6, BD Bioscience) and anti-CD69-PE-Dazzle (clone FN50, Biolegend) for surface antigens; and anti-T-bet-eFlour610 (clone 4B10, eBioscience) and anti-Eomes-PE-eFlour610 (clone Dan11mag, eBioscience) for intranuclear antigens; anti-IFNγ-V450 (BD Bioscience), anti-MIP-1β-PerCP-Cy5.5 (cloneD21–1351, BD Bioscience), anti-GM-CSF-PE-CF594 (clone BVD2–21C11, BD Bioscience), anti-TNF-FITC (clone MAb11, BD Bioscience), anti-Granzyme B-AlexaFlour700 (clone GB11, BD Bioscience) and anti-Perforin-PerCP-Cy5.5 (clone dG9, Biolegend) for intracellular antigens.

### Flow cytometric analysis

PBMC and IHL were washed with PBS before staining with live/dead fixable dye (Invitrogen) at 4 °C for 10 min in the dark. Cells were subsequently washed with PBS before blocking Fc-Receptors using saturating concentrations of FcR blocking reagent (Miltenyi) at 4 °C in the dark. Antibodies used for surface staining were added after 10 min to the samples and incubated at 4 °C in the dark for another 30 min. Cells were then washed and fixed with BD Cytofix (BD Bioscience) for 20 min at 4 °C in the dark for samples used for the analysis of surface marker expression or with Buffer A of the BD FoxP3 Buffer Set (BD Bioscience) for 10 min at room temperature (RT) in the dark to enable analysis of the intranuclear transcription factor expression, or with BD Cytofix/Cytoperm buffer for analysis of intracellular cytokines. Once fixed, cells used for analysis of transcription factor expression were incubated in buffer C of the BD FoxP3 Buffer Set according to the manufacturer’s instructions, prior to the addition of the anti-T-bet and anti-Eomes antibodies for 30 min at RT in the dark. Finally, cells were washed twice with PBS. After fixation and permeabilization of cells used for detection of intracellular antigens, cells were washed in PBS containing 1% FBS 0.1% saponin (Sigma) prior to addition of antibodies targeting intracellular antigens mentioned above for 30 min at 4 °C in the dark. After a final wash with saponin containing buffer cells were resuspended in PBS. All samples were acquired on a BD LSRII using BD FACSDiva8.0 (BD Bioscience) and data analysed using FlowJo 8 (TreeStar).

### Stimulation of intrahepatic cells

Frozen perfusate cells were thawed, washed and incubated in RPMI + 10% FCS in the presence of 10 U/ml DNAse (Roche) for 45 min at 37 °C. After washing, cells were cultured with either 5 ng/ml IL-12 and 50 ng/ml IL-18 (both R&D), 3 ng/ml PMA and 100 ng/ml Ionomycin (both Sigma, low PMA+Ionomycin) or 25 ng/ml PMA and 1 μg/ml Ionomycin for 4 h at 37 C. Anti-CD107a-APC (clone H4A3, BD Bioscience) was present during culture. Brefeldin A (Sigma) and Monensin (BD Bioscience) were added for the last 3 h of stimulation.

### Sample analysis

Data are presented as individual data points with the mean indicated for each group. Prism 5 (GraphPad Software) was used for all statistical analysis as follows: the Mann-Whitney U-test was used to compare two unpaired sample groups, the Wilcoxon-test was used to compare two paired groups, Friedmans with Dunn’s multiple comparision test was used to compare three paired samples groups and Kruskal-Wallis with Dunn’s multiple comparison test was used to compare three unpaired sample groups. Spearman’s correlation was used for the correlation of frequency of T-bet^lo^Eomes^hi^ and CXCR6 expression. *p-value < 0.05, **p-value < 0.01, ***p-value < 0.001, ****p-value < 0.0001.

## Additional Information

**How to cite this article**: Stegmann, K. A. *et al*. CXCR6 marks a novel subset of T-bet^lo^Eomes^hi^ natural killer cells residing in human liver. *Sci. Rep.* **6**, 26157; doi: 10.1038/srep26157 (2016).

## Figures and Tables

**Table 1:** Characteristics of healthy controls and patients from whom PBMC and liver tissue were obtained.

		**Healthy PBMC (n = 20)**	**Healthy liver (n = 24)**	**CHB (PBMC/liver) (n = 30)**
Age (years)	Media (range)	31 (20–40)	44 (16–69)	34 (23–59)
Sex	Female	14	5	9
	Male	6	8	21
	Unknown	–	11	
Ethnicity	Afro-Caribbean	–	–	9
	Arab	1	–	–
	Asian	1	3	10
	Caucasian	16	9	5
	South-Asian	2	–	6
	Unknown	–	12	–
ALT (IU/L)	Median (range)	n.a.	n.a.	35 (10–452)
HBV-DNA (IU/ml)	Median (range)	n.a.	n.a.	11631 (BLQ - 9.8 × 10^8^)
HBeAg+		n.a.	n.a.	8

n.a. non applicable. BLQ below limit of quantification.
